# *Curcumol* alleviates endometriosis via correcting the aberrant activation of Fas/RIPK1-mediated PANoptosis

**DOI:** 10.3389/fphar.2025.1691279

**Published:** 2025-11-18

**Authors:** Weiming Ni, Xiaoqin Wu, Li Feng, Min Xu, Yingzhou Tian

**Affiliations:** 1 Second Clinical Medical College, Guangzhou University of Chinese Medicine, Guangzhou, China; 2 Foresea Life Insurance Shaoguan Hospital, Shaoguan, China; 3 Science and Technology Innovation Center, Guangzhou University of Chinese Medicine, Guangzhou, China

**Keywords:** curcumol, endometriosis, inflammatory PANoptosis, gene expression omnibus, molecular dynamics simulations

## Abstract

Endometriosis (EMs) is an estrogen-dependent chronic inflammatory disease. While apoptosis resistance (evidenced by Bcl-2 upregulation and Bax/caspase-3 downregulation) remains a hallmark of EMs, recent studies reveal a paradoxical coexistence of aberrant apoptotic activation (Fas/FADD/Caspase-8) and inflammatory PANoptosis in ectopic lesions, suggesting novel therapeutic targets for this complex disorder. GEO database mining revealed PANoptosis-related genes in EMs, which was experimentally validated through clinical and animal studies demonstrating the critical role of Fas/FADD/Caspase-8/RIPK1 signaling. Curcumol, the active component of Curcuma zedoaria rhizomes, exhibited strong binding affinity to Fas/RIPK1 in molecular docking and dynamics simulations, and effectively alleviated EMs progression by modulating this pathway, providing novel therapeutic insights for EMs management.

## Graphical Abstract

**Figure:**
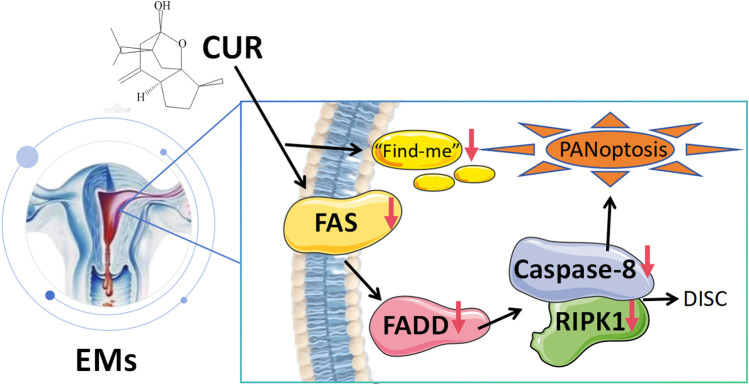

## 1 Introduction

Endometriosis (EMs), a chronic estrogen-dependent condition, involves the growth of functional endometrial tissue beyond the uterine cavity (Frackiewicz, 2000; Crump et al., 2024). EMs is characterized by pelvic pain, infertility, and ectopic endometrial implants (Shafrir et al., 2018). While retrograde menstruation occurs in most women, only ∼10% develop EMs (Griffith et al., 2010), implicating additional pathogenic drivers such as immune dysregulation, aberrant apoptosis, and pro-inflammatory mediators (Zondervan et al., 2020). The disease disproportionately impacts fertility, affecting 30%–50% of infertile women, and is frequently associated with chronic pelvic pain (Smolarz et al., 2021). Despite its benign classification, EMs exhibits malignant-like behaviors, including invasive progression and high recurrence rates (Steinbuch et al., 2024). Diagnostic challenges persist, with average delays of 8–10 years due to symptom overlap with other gynecological conditions (Greene et al., 2009). Current research prioritizes non-invasive biomarkers to complement laparoscopic confirmation (Ahn et al., 2017), while mechanistic studies explore genetic, hormonal, and microenvironmental contributions (Burney and Giudice, 2019; Taylor et al., 2021). Current therapies (e.g., hormonal suppression and surgery) face challenges of recurrence and side effects (Becker et al., 2022; Cullen et al., 2013), underscoring the need for novel therapeutic strategies.

Apoptosis, a tightly regulated form of programmed cell death, serves as a fundamental mechanism for removing dysfunctional or superfluous cells while preserving tissue homeostasis (Zhong et al., 2024). Its impairment contributes to numerous pathological conditions, ranging from neurodegeneration and ischemia to malignancies and autoimmune disorders (Elmore, 2007; Bertheloot et al., 2021). In the context of endometriosis (EMs), apoptotic clearance of both ectopic and eutopic endometrial cells prevents necrotic aggregation and aberrant implantation (Agic et al., 2009; Kobayashi et al., 2024). Notably, the eutopic endometrium in EMs patients exhibits reduced pro-apoptotic activity and enhanced anti-apoptotic signaling compared to healthy controls (Harada et al., 2007), potentially enabling refluxed cells to evade elimination and establish ectopic lesions. Besides, dysregulated cell death pathways have emerged as key pathogenic drivers. While apoptosis resistance via Bcl-2/Bax imbalance is established (Mclaren et al., 1997; Zhang et al., 2025), recent studies report paradoxical upregulation of pro-death receptors like Fas in ectopic lesions (Garcia-Velasco et al., 2002; Sturlese et al., 2011), suggesting complex death signaling alterations. Critically, RIPK1, which is a master regulator of inflammatory cell death, is elevated in EMs (Tian et al., 2021a), yet its spatial co-expression with Fas and targetability by natural compounds remain unexplored. Elucidating this specific mechanism represents a novel strategic opportunity to disrupt the disease’s core apoptosis-inflammation cycle, moving beyond mere symptom suppression.

Plant-derived natural metabolites have gained prominence in pharmaceutical research due to their multi-target therapeutic potential (Meresman et al., 2021). These bioactive compounds demonstrate significant efficacy against endometriosis (EMs) through diverse pathways, particularly by enhancing apoptosis, suppressing inflammation, inhibiting angiogenesis, and counteracting oxidative stress. Clinical evidence from systematic reviews supports their favorable safety profile and therapeutic value, making them ideal candidates for complementary medicine (Xu et al., 2022). Notably, traditional herbal medicines offer distinct advantages for chronic EMs management, including cost-effectiveness and minimal adverse effects compared to conventional drugs.

Curcumol (CUR), a primary bioactive terpenoid from Curcuma zedoaria (Ezhu), demonstrates efficacy in EMs animal models by reducing lesion size and inflammation (Lu et al., 2024; Tian et al., 2021b). Which can suppress the JAK2/STAT3 signaling cascade, thereby attenuating inflammatory cytokine production in ectopic endometrial stromal cells. Additionally, they demonstrate potent anti-proliferative and anti-migratory effects, ultimately leading to regression of ectopic lesions (Wang et al., 2022). Recent studies demonstrate that CUR regulates cell death through multiple interconnected mechanisms. It induces tumor cell apoptosis via the IGF-1R and p38 MAPK pathways while concurrently triggering autophagic cell death through JNK signaling activation (Wang et al., 2015; Zhang and Wang, 2017). Furthermore, CUR promotes programmed necrosis by activating Sirt1-mediated Atg5 deacetylation, which enhances protein-protein interactions and stimulates autophagy (Sun et al., 2022). However, the precise mechanisms underlying curcumol’s therapeutic potential in endometriosis (EMs) remain elusive. To address this knowledge gap, our study was designed to investigate two key questions: Whether Fas/RIPK1 co-overexpression occurs in human EMs lesions; Whether CUR can directly engage Fas/RIPK1 to modulate their activity, providing novel therapeutic insights for EMs management.

## 2 Materials and methods

### 2.1 Principal reagents

CUR (purity ≥98%) was purchased from Shanghai Ziyi Bio-Technology Co., Ltd. (Shanghai, China). All other chemicals, unless specified, were purchased from Sigma-Aldrich.

### 2.2 Gene Expression Omnibus (GEO)

A comprehensive evaluation was conducted on all datasets from the Gene Expression Omnibus (GEO), an NCBI-curated repository of high-throughput genomic expression data, with particular attention to EMs-related studies. For inclusion in our analysis, each study had to fulfill two key criteria: (1) Documentation of the experimental platforms and methodologies employed. (2) Use of normal samples as controls.

### 2.3 Animals

Female C57BL/6 mice (6–8 weeks old, 18–20 g) were obtained from Guangdong Medical Laboratory Animal Center (group size: n = 6 unless stated otherwise). Animals were housed under specific pathogen-free (SPF) conditions with a 12 h light/dark cycle and provided *ad libitum* access to food and water. Subsequent to a 7-day acclimatization period, a daily pretreatment with estradiol (0.5 mg/kg, administered by gavage) was commenced 1 week before model induction. On day 8, all subjects were subjected to surgery under anesthesia via intraperitoneal injection of 2% sodium pentobarbital (50 mg/kg). The ectopic endometrial model (MOD) was surgically induced by grafting a 5 × 5 mm endometrial tissue section onto the peritoneal wall; control (CON) animals received a sham operation. The estradiol supplementation was continued postoperatively via daily gavage at the identical dosage. After 3 weeks, Ectopic mice were randomized into two cohorts: untreated (MOD) and Curcumol-treated (CUR, 30 mg/kg) (Tian et al., 2021a). Throughout the experiment, all groups were maintained on standard chow. Control and MOD animals received daily saline via oral gavage, while the CUR group was administered the therapeutic regimen for the subsequent 4 weeks. The study complied with ethical guidelines approved by Guangzhou University of Chinese Medicine’s Animal Ethics Committee (No. 20241204013).

### 2.4 Histopathological examination ((H&E)) and immunofluorescence evaluation

Uterine and parietal peritoneal tissues were fixed in 4% paraformaldehyde (24 h) for histopathological processing. Following dehydration and paraffin embedding, serial sections (5 μm) were prepared for H&E staining. Parallel samples of uterine tissue were cryoprotected in 30% sucrose (48 h), embedded in OCT compound, and sectioned at identical thickness for immunofluorescence studies. Tissue sections were probed with primary antibodies against FAS and RIPK (Servicebio, China) at 4 °C overnight. Digital imaging was performed using a standardized scanner (KONFOONG BIOINFORMATION TECH CO., LTD., China).

### 2.5 Western blot (WB) analysis

Uterine tissue protein extracts were homogenized in RIPA buffer, with concentrations determined by BCA assay (Beyotime Biotechnology). Equal protein aliquots (80–100 μg) were separated via 10% SDS-PAGE and electrotransferred to PVDF membranes. Immunoblotting was conducted using primary antibodies against FAS, FADD, Caspase-8, RIPK1, and p-RIPK1 (Proteintech), with β-actin (Abclonal) as loading control. Protein bands were visualized and quantified using Image Lab v4.0.

### 2.6 Molecular docking analysis

Following established protocols (Li et al., 2024), molecular docking was conducted to assess potential interactions between bioactive compounds and MS-associated targets. Molecular docking studies were performed using AutoDock Vina (version 1.2.0) to predict the binding mode of CUR with FAS and RIPK1. The crystal structures of FAS (Uniprot ID: P49327) and RIPK1 (Uniprot ID: P25445) were obtained from the Uniprot. Receptor proteins were prepared by removing crystallographic water molecules, adding hydrogen atoms, and assigning Gasteiger charges using AutoDockTools. The ligand CUR was energy-minimized with the MMFF94 force field.

Docking grids were centered on the active site residues of FAS (center coordinates: x = 27.5631, y = 21.5840, z = 49.9819 Å; box size: 20 × 20 × 20 Å^3^) and RIPK1 (center coordinates: x = −13.5856, y = −7.3611, z = −20.6319 Å; box size: 20 × 20 × 20 Å^3^), ensuring complete coverage of the binding pockets and adjacent residues. For each run, a maximum of 10 docking poses were generated, and conformations with binding energies lower than −6.0 kcal/mol were considered significant. The top-ranked docking poses were selected for further molecular dynamics simulations.

### 2.7 Molecular dynamics (MD) simulations

MD simulations were carried out using GROMACS (version 2024.5) with the CHARMM36 force field. Each protein–ligand complex was solvated in a cubic TIP3P water box with at least 1.0 nm distance from the protein to the box edge. Counter ions (Na^+^/Cl^−^) were added to neutralize the system. Energy minimization was performed using the steepest descent algorithm, followed by equilibration under NVT (100 ps) and NPT (100 ps) ensembles.

The production MD simulations were run for 100 ns at a constant temperature of 300 K and pressure of 1 atm using the velocity rescale thermostat and Parrinello–Rahman barostat. A cutoff of 1.0 nm was applied for both van der Waals and Coulomb interactions, and long-range electrostatics were treated with the Particle Mesh Ewald (PME) method. Trajectory data were collected every 10 ps and analyzed for root-mean-square deviation (RMSD), solvent-accessible surface area (SASA), and free energy landscape (FEL) to evaluate the stability and conformational dynamics of the complexes.

### 2.8 Statistical analysis

Data are expressed as mean ± SEM. For statistical evaluation, GraphPad Prism 9.0 (San Diego, CA, United States) was employed, utilizing one-way ANOVA followed by Dunnett’s *post hoc* test for multiple comparisons. A P-value below 0.05 was considered statistically significant.

## 3 Results

### 3.1 Bioinformatic analysis of GEO datasets was performed to examine differential gene expression and predict KEGG pathway alterations in endometriosis

**FIGURE 1:**
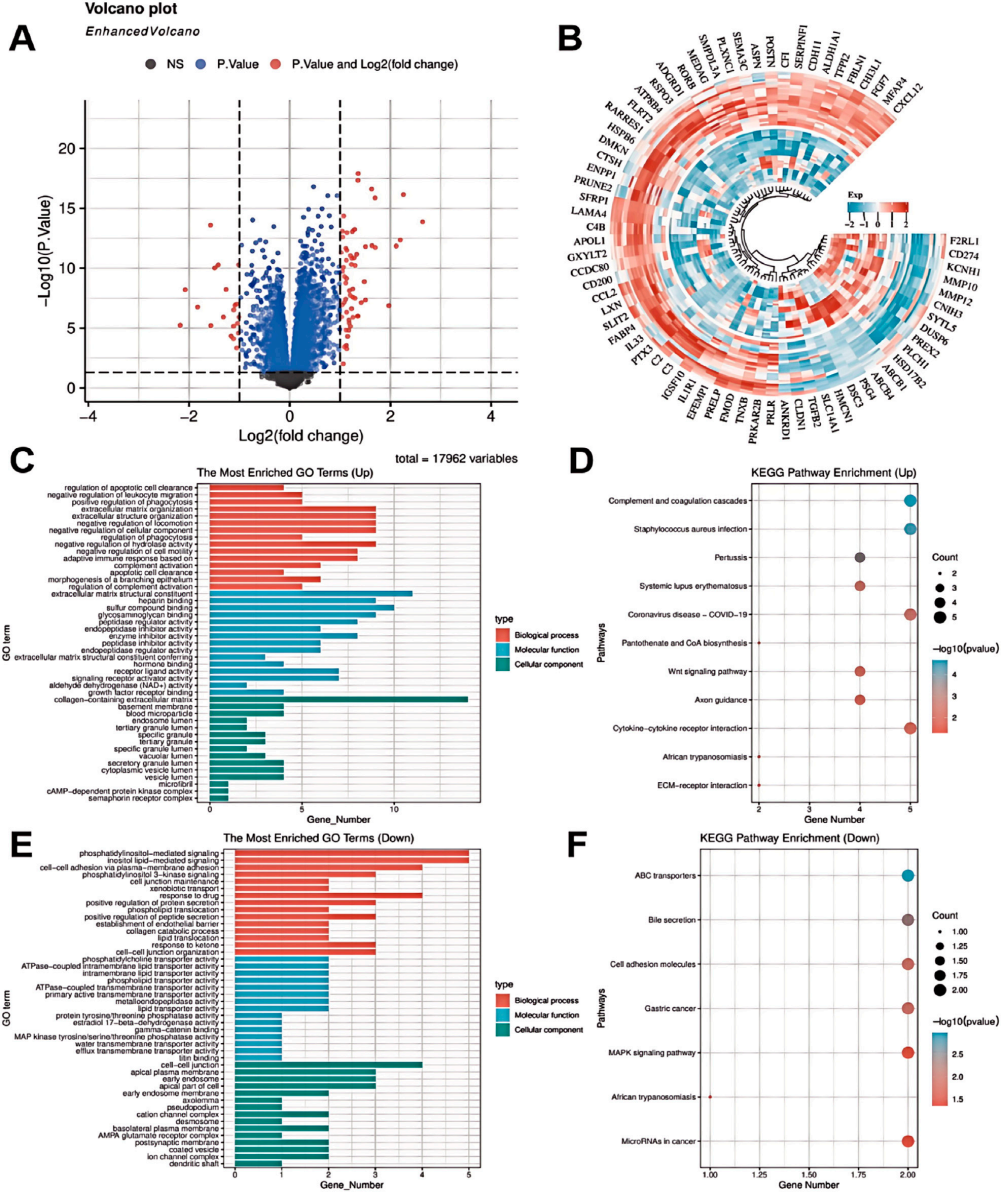
Bioinformatic analysis of GEO datasets was performed to examine differential gene expression and predict KEGG pathway alterations in endometriosis. **(A)** Volcano plot of differentially expressed genes. **(B)** Heatmap of differentially expressed genes. **(C)** GO enrichment analysis of upregulated genes. **(D)** KEGG pathway enrichment of upregulated genes. **(E)** GO enrichment analysis of downregulated genes. **(F)** KEGG pathway enrichment of downregulated genes.

Differential gene expression analysis between endometriosis (EMs) and normal endometrial tissue samples was performed through data mining (Figure 1). Our analysis revealed significant upregulation of ADGRD1, FGF7, and ATP8B4 in EMs, potentially associated with aberrant cellular proliferation, immune responses, and tissue repair processes. Notably, elevated FGF7 expression may contribute to angiogenesis and tissue remodeling, thereby promoting ectopic endometrial growth. Conversely, genes including MMP10 and IL33 showed marked downregulation, possibly linked to impaired cell migration, immune evasion, and suppressed tissue repair mechanisms (Figures 1A,B). GO enrichment analysis demonstrated that upregulated genes were predominantly involved in negative regulation of cell migration and extracellular matrix reorganization, while downregulated genes were enriched in cell membrane adhesion and phosphatidylinositol signaling pathways. These findings suggest that EMs pathogenesis involves dysregulated immune responses and altered cell-cell interactions (Figures 1C,E).

KEGG pathway analysis further identified significant enrichment of these differentially expressed genes in critical pathways including complement and coagulation cascades, systemic lupus erythematosus, and Wnt signaling (Figure 1D). Pathway activation may play pivotal roles in EMs pathophysiology, particularly in modulating immune responses and cellular migration. Mechanistically, upregulated genes (FGF7, ATP8B4) may facilitate lesion expansion through enhanced cell survival, proliferation, and angiogenesis. Conversely, downregulation of immune-related genes (e.g., IL33) could promote immune evasion, enabling lesion persistence. Reduced MMP10 expression might impair matrix degradation, potentially disrupting tissue repair and exacerbating cellular damage. Notably, KEGG analysis highlighted Wnt and MAPK signaling pathway enrichment, suggesting their involvement in PANoptosis regulation via cell survival, proliferation, and migration control. MAPK pathway dysregulation may particularly disrupt the balance between cellular responses, apoptosis, and inflammatory reactions, contributing to EMs-associated tissue damage (Figure 1F). Collectively, these findings implicate PANoptosis as a potentially crucial mechanism in EMs progression.

### 3.2 RIPK1 as a central mediator of PANoptosis in endometriosis lesions

**FIGURE 2:**
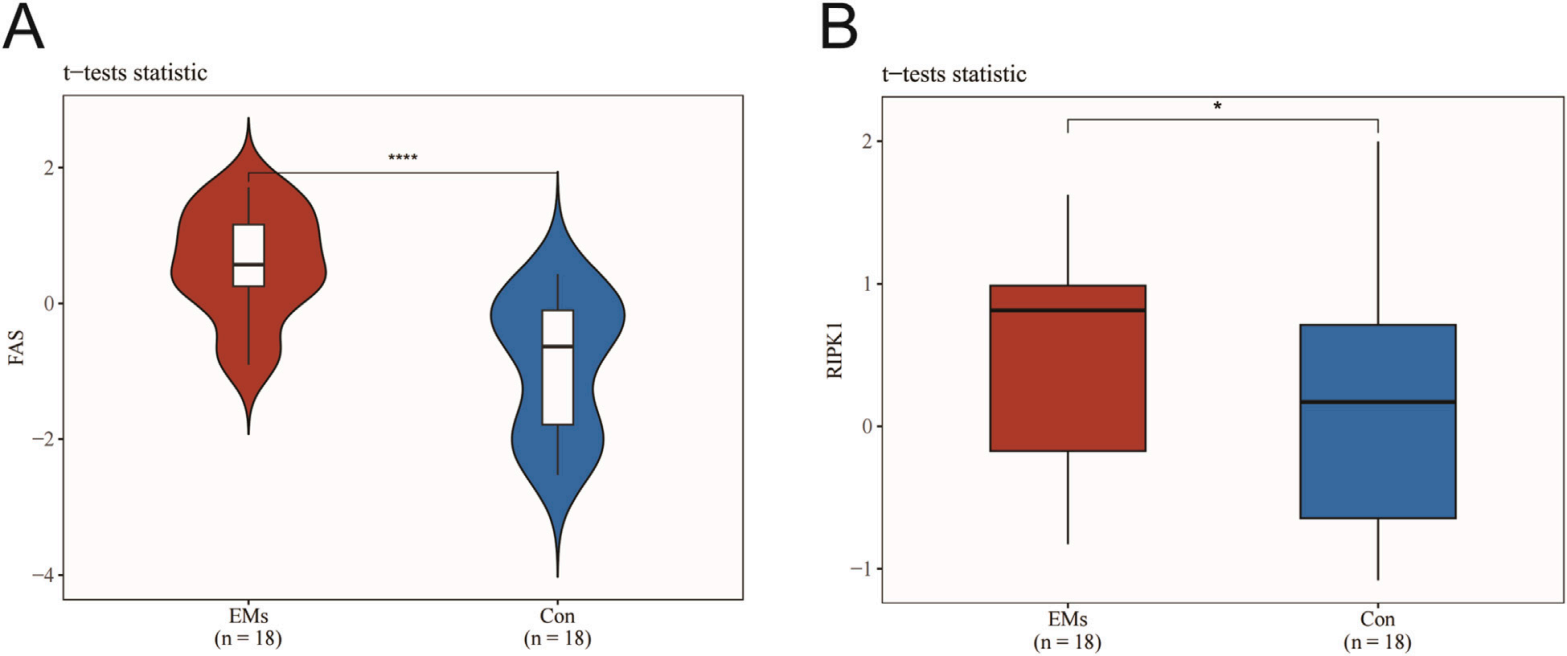
RIPK1 as a central mediator of PANoptosis in endometriosis lesions. **(A)** Fas expression levels. **(B)** RIPK1 expression levels. Data are presented as mean ± SEM (n = 18). ^*^ *P < 0.05*, ^**^ *P < 0.01*, ^***^ *P < 0.001*, ^****^ *P < 0.0001* vs. the model group.

Under normal physiological conditions, Fas and RIPK1 expression facilitates cellular responses to damage and stress by initiating apoptotic or necrotic pathways to eliminate compromised cells. However, in endometriosis (EMs), we observed significantly elevated expression levels of both Fas and RIPK1 compared with normal controls (CON). This aberrant overexpression may promote PANoptosome complex assembly and exacerbate inflammatory responses. Statistical analysis using Student’s t-test revealed highly significant differences in Fas (P < 0.0001) and RIPK1 (P < 0.05) expression between EMs and normal endometrial tissues. These findings suggest that RIPK1 not only plays a pivotal role in PANoptosis regulation but may also serve as a potential therapeutic target for modulating immune evasion and impaired tissue repair mechanisms in EMs pathogenesis (Figures 2A,B).

### 3.3 The FAS/RIPK1 signaling axis emerges as a pivotal regulator in endometriosis pathogenesis

**FIGURE 3:**
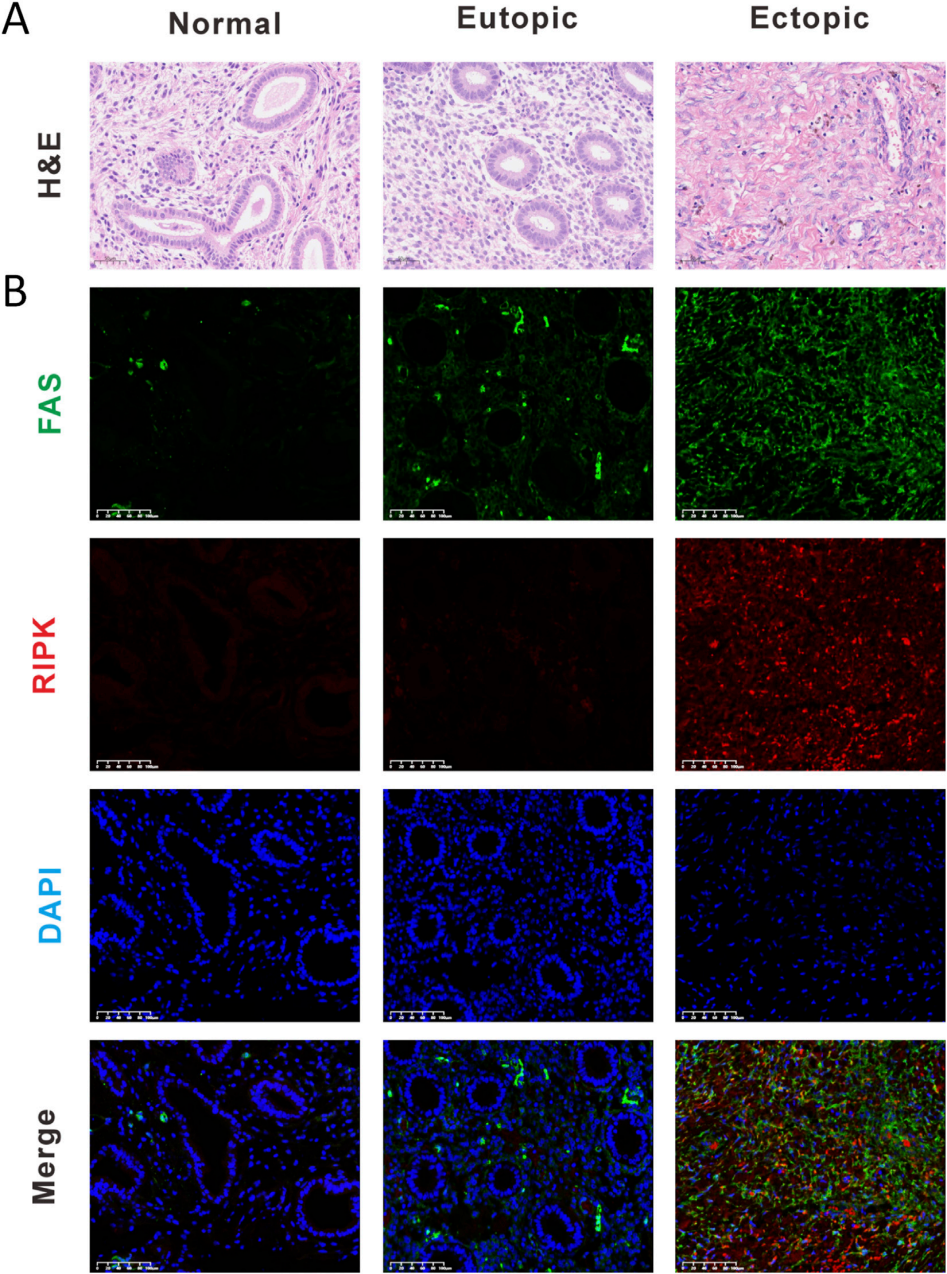
The FAS/RIPK1 signaling axis emerges as a pivotal regulator in endometriosis pathogenesis. **(A)** Representative H&E staining images (Scale bar = 50 μm). **(B)** Representative immunofluorescence (Scale bar = 100 μm). (IF) images of normal endometrium (Normal), eutopic endometrium from EMs patients (Eutopic), and ectopic endometrial lesions (Ectopic) (Scale bar = 100 μm).

This study was approved by the Ethics Committee of Guangdong Provincial Hospital of Chinese Medicine (Approval No. ZE2025-098-01). We collected three groups of clinical specimens (n = 9): normal controls (Normal), eutopic endometrium from EMs patients (Eutopic), and ectopic endometrial lesions (Ectopic). Specimen quality was first verified by H&E staining to confirm proper endometrial sampling. Using immunofluorescence double-staining, we examined the expression and co-localization patterns of FAS and RIPK1. Notably, ectopic endometrial lesions exhibited significantly higher expression levels of both FAS and RIPK1 compared to normal controls. A similar trend of elevated FAS expression was observed in eutopic endometrium from EMs patients. These findings strongly implicate the FAS/RIPK1 signaling axis in the pathogenesis of endometriosis (Figures 3A,B).

### 3.4 CUR effectively inhibits endometriosis progression by modulating the Fas/FADD/caspase-8/RIPK1 signaling pathway

**FIGURE 4:**
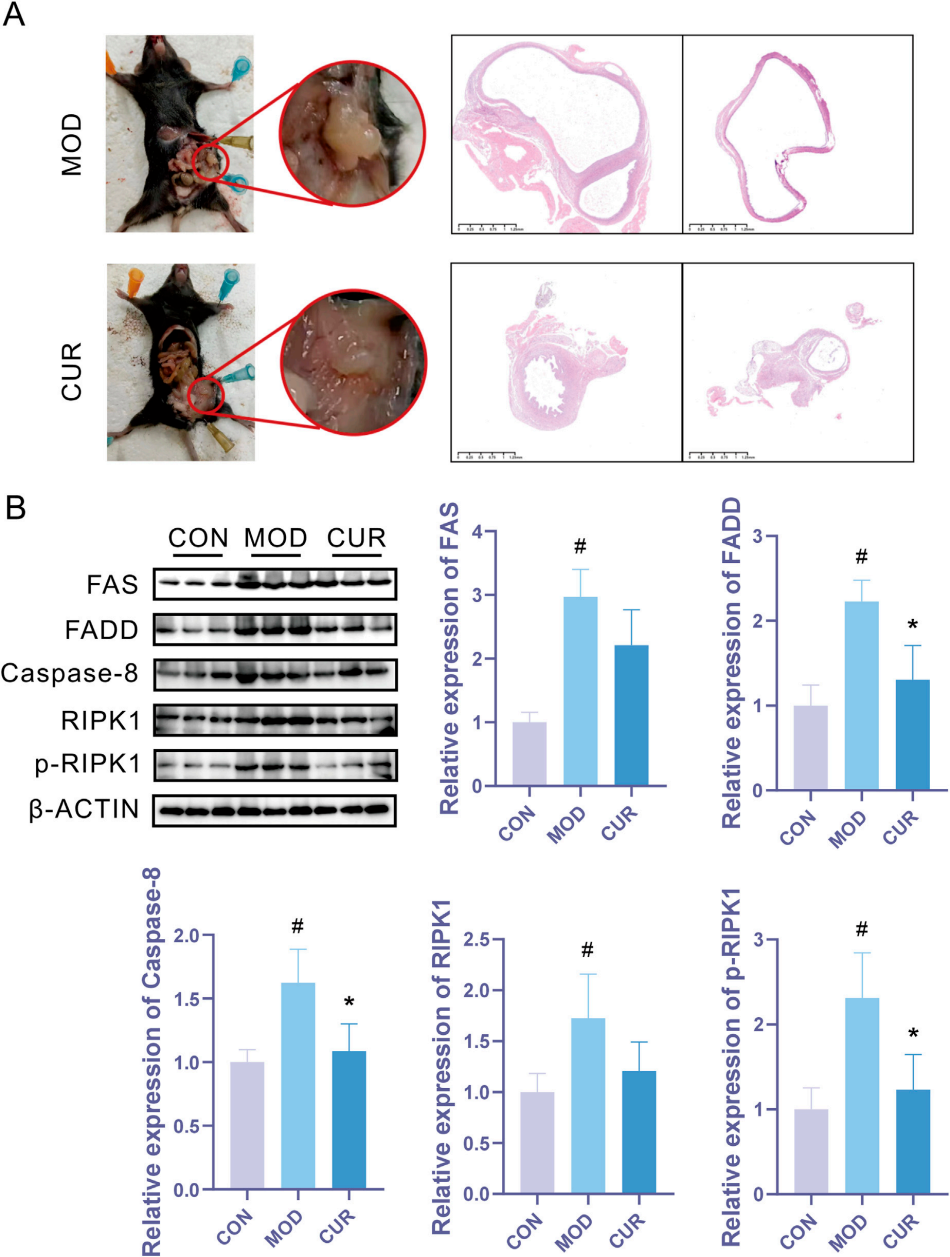
CUR effectively inhibits endometriosis progression by modulating the Fas/FADD/Caspase-8/RIPK1 signaling pathway. **(A)** Representative macroscopic images of lesions and corresponding H&E-stained histological sections. **(B)** Western blot analysis of Fas/FADD/Caspase-8/RIPK1 pathway protein expression (n = 3 biological replicates). Data are presented as mean ± SEM. ^*^ *P* < 0.05, ^**^ *P* < 0.01, ^***^ *P* < 0.001, ****P < 0.0001 vs. the model group. ^#^ *P* < 0.05, ^##^ *P* < 0.01, ^###^ *P* < 0.001, ^*####*^ *P < 0.0001* vs. the control group.

To evaluate the therapeutic potential of CUR in endometriosis, we established an EMs animal model to investigate it's *in vivo* efficacy. CUR treatment significantly attenuated disease progression, as evidenced by reduced lesion size (Figure 4A). Immunoblot analysis revealed marked upregulation of Fas/FADD/Caspase-8/RIPK1 pathway components in the model group compared with controls, which was effectively normalized by CUR administration (Figure 4B). These results suggest that CUR exerts its anti-endometriotic effects through modulation of the Fas-mediated apoptotic pathway.

### 3.5 Molecular docking and molecular dynamics (MD) simulations of CUR with FAS and RIPK1

**FIGURE 5:**
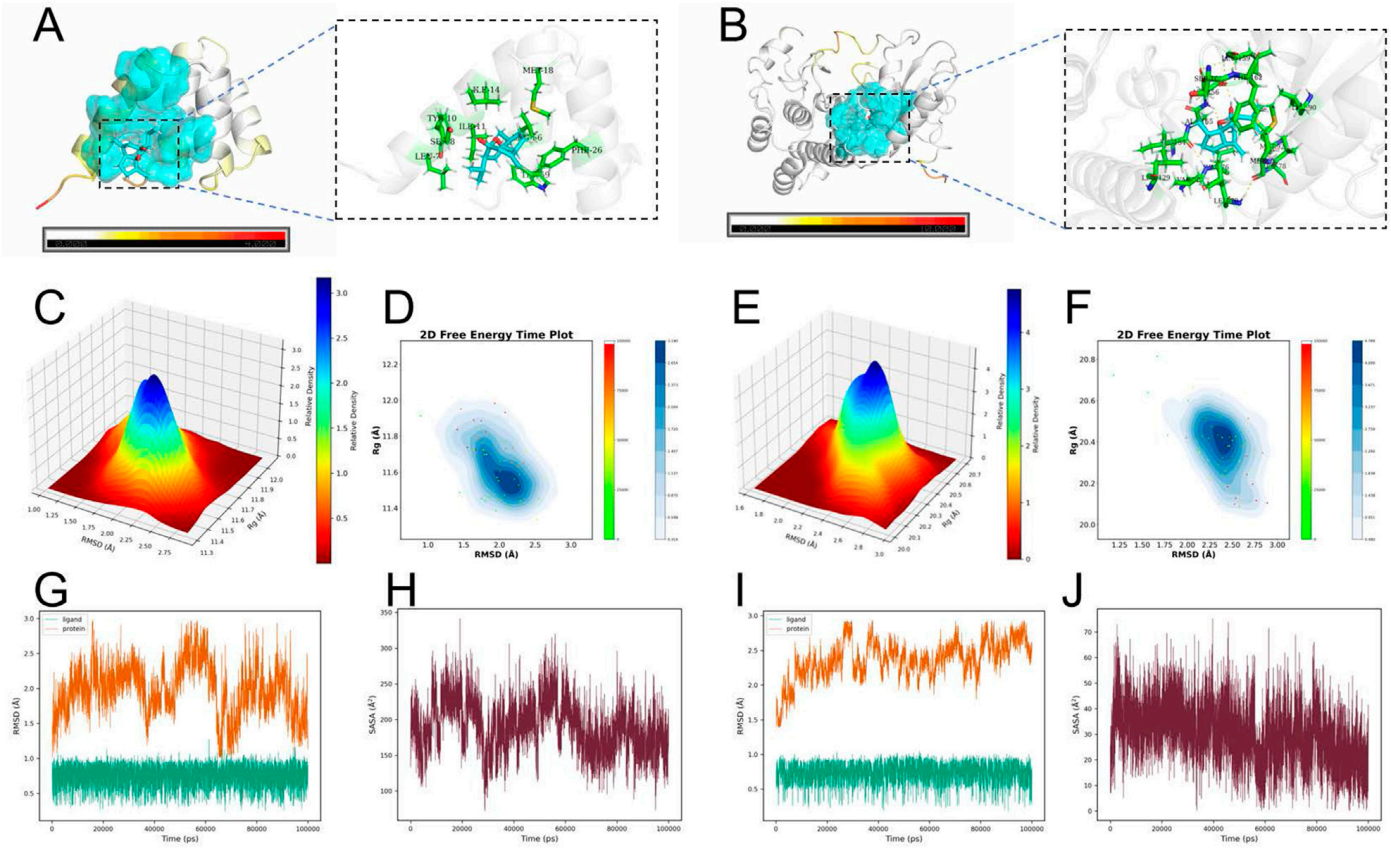
Molecular docking and molecular dynamics (MD) simulations of CUR with FAS and RIPK1. **(A)** Predicted binding mode of CUR with FAS. **(B)** Predicted binding mode of CUR with RIPK1. **(C)** Three-dimensional free energy landscape (FEL) of the FAS–CUR complex. **(D)** Two-dimensional free energy profile of the FAS–CUR complex over simulation time. **(E)** Three-dimensional FEL of the RIPK1–CUR complex. **(F)** Two-dimensional free energy profile of the RIPK1–CUR complex over simulation time. **(G)** Root-mean-square deviation (RMSD) trajectory of the FAS–CUR complex. **(H)** Solvent-accessible surface area (SASA) analysis of the FAS–CUR complex. **(I)** RMSD trajectory of the RIPK1–CUR complex. **(J)** SASA analysis of the RIPK1–CUR complex. Docking was performed using AutoDock Vina with grid boxes centered at the active sites of FAS (27.5631, 21.5840, 49.9819 Å; 20 × 20 × 20 Å^3^) and RIPK1 (−13.5856, −7.3611, −20.6319 Å; 20 × 20 × 20 Å^3^). MD simulations were carried out in GROMACS (100 ns, CHARMM36 force field, TIP3P water, 300 K, 1 atm) to validate the structural stability and thermodynamic properties of the complexes.

To investigate the interactions between CUR and FAS/RIPK1, molecular docking was performed. The results demonstrated that CUR stably binds to the active sites of both FAS and RIPK1, forming multiple hydrogen bonds and hydrophobic interactions. In the FAS-CUR complex (Figure 5A), CUR established a stable hydrogen-bonding network with key residues, including LEU-7 and MET-18. Similarly, in the RIPK1-CUR complex (Figure 5B), CUR exhibited favorable interactions with LEU-159 and PHE-162. Furthermore, binding free energy calculations revealed strong binding affinities, with ΔG values of −7.7 kcal/mol (FAS-CUR) and −8.7 kcal/mol (RIPK1-CUR), indicating high thermodynamic stability of these complexes.

To further evaluate the stability of CUR-FAS and CUR-RIPK1 complexes, we performed 100-ns molecular dynamics (MD) simulations. The structural dynamics were assessed through root-mean-square deviation (RMSD) analysis, solvent-accessible surface area (SASA) calculations, and free energy landscape (FEL) profiling. Both complexes achieved stable conformations after 80 ns of simulation, with average RMSD values of 1.9842 Å for FAS-CUR and 2.3666 Å for RIPK1-CUR, indicating robust structural integrity of the bound complexes (Figures 5G–I). The 3D FEL analysis revealed minimum free energy states at −18.4762 kcal/mol (FAS-CUR) and −32.0261 kcal/mol (RIPK1-CUR). The 2D free energy time evolution profiles demonstrated predominant population of low-energy states, confirming thermodynamically favorable binding of CUR to both targets (Figures 5C–F). SASA analysis showed average values of 189.46 Å^2^ for FAS-CUR and 29.86 Å^2^ for RIPK1-CUR, suggesting optimal ligand exposure that facilitates stable protein-ligand interactions while maintaining binding pocket occupancy (Figures 5H,J).

## 4 Discussion

Endometriosis (EMs) is an estrogen-dependent chronic inflammatory disease characterized by pelvic pain, infertility, and refractory clinical course, representing a significant challenge in gynecology. While its pathogenesis remains incompletely understood, emerging evidence highlights the interplay of immune dysfunction (including impaired macrophage phagocytosis, NK cell deficiency, and Treg-mediated immunosuppression), chronic inflammation (mediated by NLRP3/IL-1β/NF-κB axis), oxidative stress (with elevated ROS and mitochondrial dysfunction), and dysregulated cell death pathways (Zondervan et al., 2020).

Studies have revealed significant impairments in both innate and adaptive immunity in EMs patients. Specifically, Symons et al. (2018) demonstrated compromised phagocytic function of peritoneal macrophages in clearing ectopic endometrial tissue, while Fan et al. (2023) reported impaired immune surveillance by NK cells. Additionally, increased Treg infiltration in ectopic lesions contributes to immunosuppression (Zhang et al., 2018), and Wu et al. (2017) identified defective antigen presentation by dendritic cells, leading to impaired T cell activation.

A hallmark of EMs is sustained inflammation, characterized by elevated levels of proinflammatory mediators (NLRP3, caspase-1, and IL-1β) in ectopic lesions, which promote cell proliferation, angiogenesis, and fibrosis. This inflammatory cascade is further amplified by hyperactivated NF-κB signaling (Al et al., 2024), though NLRP3 inhibitors show therapeutic potential by suppressing IL-1β release (Murakami et al., 2022). Moreover, EMs patients exhibit marked oxidative stress (Cordaro et al., 2021), where excessive ROS production induces mitochondrial dysfunction and activates pathological cell death pathways (Scutiero et al., 2017). We now emphasize that while inhibiting NLRP3 or NF-κB alleviates inflammation, targeting Fas/RIPK1 simultaneously disrupts the vicious cycle that drives both lesion survival and the inflammatory microenvironment, offering a more fundamental therapeutic strategy.

Although classical apoptosis resistance (evidenced by Bcl-2 upregulation and Bax/caspase-3 downregulation) remains a hallmark (Taghavipour et al., 2020), recent studies reveal a paradoxical coexistence of aberrant apoptotic activation (Fas/FADD/Caspase-8) and inflammatory PANoptosis in ectopic lesions, where DAMPs release exacerbates local inflammation, suggesting novel therapeutic targets for this complex disorder (Cullen et al., 2013). The “Find-me” signals in endometriosis pathogenesis and therapeutic potential. Known “Find-me” signal molecules, including chemokines such as CX3CL1, are recognized by immune cells through specific receptors to mediate their migration toward dying cells (Ravichandran, 2010). During apoptotic clearance, chemokines serve as PS (phosphatidylserine)-bound “Find-me” signals. Apoptotic cells upregulate surface-exposed anionic PS while downregulating anionic glycosaminoglycans (GAGs). The PS-bound chemokines directly activate chemokine receptors, initiating a sequential process of immune cell chemotaxis, functional polarization, and apoptotic clearance/inflammatory regulation that maintains tissue homeostasis under physiological conditions (Pontejo and Murphy, 2021). However, in endometriosis (EMs) lesions, aberrant activation of the Fas pathway may cause excessive “Find-me” signal release, leading to macrophage over-recruitment and sustained inflammation. Studies demonstrate that RIPK1 hyperactivation in EMs lesions potentially releases DAMPs (e.g., HMGB1) through necroptosis, which recruits CCR2+ macrophages and activates the NLRP3 inflammasome, creating a vicious “death-inflammation” cycle (Clucas and Meier, 2023; Rodriguez et al., 2022). Therefore, targeted inhibition of excessive Fas/RIPK1 pathway activation - by reducing Fas/FADD/Caspase-8/RIPK1-PANoptosome complex assembly, decreasing abnormal “Find-me” signal release, and limiting downstream immune cell recruitment - may serve as a key therapeutic strategy to disrupt the vicious “apoptosis signal-inflammatory feedback” cycle in endometriosis.

Curcuma zedoaria (Christm.), a traditional Chinese medicinal plant from the Zingiberaceae family, is clinically employed for endometriosis (EMs) treatment. Its serum-containing drugs demonstrate inhibitory effects on EMs cellular pathology, with CUR being identified as one of the primary bioactive constituents (Wang et al., 2023). Network pharmacology and experimental studies reveal that Curcuma zedoaria-containing herbal formulas exert multi-target therapeutic effects in EMs. For instance, the Extrauterine Pregnancy Formula No. 2 (containing C. zedoaria and Sparganium stoloniferum) reduces proinflammatory cytokine secretion (TNF-α, IL-6) while promoting Caspase-3-mediated apoptosis, thereby suppressing ectopic endometrial angiogenesis and lesion proliferation (Yu and Wang, 2024). Animal studies confirm that C. zedoaria monotherapy significantly decreases ectopic lesion volume in EMs rats, mechanistically linked to Bax/Bcl-2 apoptotic pathway modulation (Han et al., 2019). Notably, the Sparganium-Curcuma herb pair exhibits synergistic anti-EMs effects superior to single-agent treatment (Qin et al., 2022), evidenced by a 46.7% reduction in peritoneal IL-1β levels compared to the model group and molecular docking-confirmed high binding affinity between its active component tanshinone IIA and TP53.

CUR, the primary bioactive constituent of Curcuma zedoaria, exhibits pleiotropic biological effects including anti-inflammatory, antioxidant, and antitumor activities, as well as modulation of autophagy, immune regulation, and cell death pathways. For example, Yang et al. (2021) demonstrated that CUR exerts anti-inflammatory activity by suppressing NF-κB and MAPK signaling pathways, effectively reducing pro-inflammatory cytokines (TNF-α, IL-1β) while elevating anti-inflammatory IL-10 secretion (Yuandani et al., 2021). CUR exhibits potent antioxidant capacity through multiple mechanisms: enhancing SOD and GSH-Px activities to scavenge free radicals (Sathyabhama et al., 2022), and preserving mitochondrial function to alleviate oxidative damage (Hashem et al., 2021). CUR demonstrates broad-spectrum antitumor activity through multiple mechanisms. Ning et al. (2020) revealed its ability to inhibit tumor growth by inducing apoptosis. Notably, in prostate cancer, CUR triggers both autophagy and ferroptosis while modulating lipid metabolism, with its anti-tumor effects mediated through regulation of the gut microbiota-DNMT1/IGFBP2 axis involving both immune response and metabolic reprogramming (Xu et al., 2024). Immunomodulatory effects of CUR include enhanced NK and T cell activity (Yuandani et al., 2021) and promotion of macrophage polarization toward anti-inflammatory phenotypes (Abdollahi et al., 2023). Cell death regulation involves IGF-1R/p38 MAPK-mediated apoptosis, JNK-dependent autophagic death (Zhang and Wang, 2017), and Sirt1-induced Atg5 deacetylation that enhances protein interactions to coordinate autophagy and programmed necrosis (Sun et al., 2022). In endometriosis (EMs), CUR shows therapeutic potential by suppressing inflammatory responses in the peritoneal microenvironment, as demonstrated in experimental rat models (Nie et al., 2019). These multifaceted pharmacological activities position CUR as a promising candidate for EMs treatment.

This existing evidence, combined with our findings, positions CUR as a promising candidate with a potentially superior safety profile compared to existing hormonal therapies, which are associated with side effects like weight gain, mood changes, and thromboembolic risks (Vercellini et al., 2014). Furthermore, hormonal therapies fail to eradicate lesions, and symptoms frequently recur after treatment cessation (Saunders and Horne, 2021). In contrast, our findings demonstrate that CUR directly targets the apoptosis-inflammation feedback loop within the ectopic lesions. By acting on this fundamental pathological driver, CUR may offer a more durable remission after treatment cessation, presenting a promising alternative for patients who are intolerant of or reluctant to use hormonal therapies. Surgery is effective for immediate symptom relief but is invasive, carries risks of adhesion formation, and has a high recurrence rate of up to 50% within 5 years (Guo, 2009). CUR, as a potential oral medication, could be envisioned as a neoadjuvant therapy to reduce lesion size before surgery or, more importantly, as an adjuvant therapy to prevent post-surgical recurrence by suppressing the inflammatory microenvironment that fuels regeneration. In summary, while further development is required, curcumol’s unique mechanism, which targets the core pathological circuit rather than systemic estrogen suppression, and its foundation in natural product chemistry present a compelling strategy for a more targeted and potentially better-tolerated management of endometriosis.

The primary limitation of this study lies in the relatively small size of the clinical cohort, which may constrain the statistical power and the generalizability of our findings across the broad spectrum of endometriosis phenotypes. While our results provide compelling proof-of-concept for the role of the Fas/RIPK1-PANoptosis axis. Our subsequent research will prioritize the validation of these mechanisms in a larger, multi-center patient population to enhance the robustness and clinical relevance of our findings; and the utilization of diverse animal models to comprehensively assess the therapeutic efficacy of CUR and the broader applicability of this pathway. Furthermore, while the polypharmacology of natural compounds may offer therapeutic advantages for multifactorial diseases, it complicates the exclusion of potential off-target effects. To establish a definitive causal link, future studies will incorporate proteomic profiling for systematic target identification, coupled with genetic perturbation experiments to functionally validate the pathway necessity. At the same time, our future research priorities include caspase-8 activity assays to confirm enzymatic inhibition, co-immunoprecipitation to assess PANoptosome formation disruption, and measurement of extracellular ATP/HMGB1 levels to evaluate DAMP release suppression. These investigations are essential for delineating the precise mechanism of action and advancing the translational development of CUR in endometriosis.

Despite the promising mechanistic insights, the clinical advancement of CUR is contingent upon overcoming pharmacokinetic limitations common to natural products, including poor bioavailability and stability. Our study thus serves as a foundational proof-of-concept. Subsequent research must prioritize addressing these translational hurdles via medicinal chemistry, novel formulation strategies, and rigorous ADME profiling to unlock its full therapeutic potential.

## 5 Conclusion

This study employed an integrative multi-omics approach to investigate Fas/RIPK1 pathway dysregulation in endometriosis (EMs). First, we performed transcriptomic analysis of publicly available EMs datasets from GEO to identify aberrant Fas/RIPK1 signaling. Second, tissue-level validation through immunofluorescence co-staining was conducted using clinical specimens (eutopic/ectopic endometrium) and control samples. Third, we computationally validated CUR-Fas/RIPK1 interactions using molecular docking followed by 100-ns molecular dynamics simulations. Our results demonstrated that CUR exhibits high binding affinity to Fas/RIPK1 *in silico* and effectively attenuates EMs progression by targeting this pathway, offering new mechanistic insights for EMs therapeutics.

## Data Availability

The original contributions presented in the study are included in the article/supplementary material, further inquiries can be directed to the corresponding authors.

## Abbreviations

CUR, Curcumol; DAMPs, Damage-Associated Molecular Patterns; EMs, Endometriosis; GEO, Gene Expression Omnibus; H&E, Hematoxylin and eosin; RMSO, Root-mean-square Deviation; SASA, Solvent-accessible Surface Area; FEL, Free Energy Landscape.
